# Intercalation of Neutral Guests in Pillared Salt Cocrystals of 5-Ureidosalyclic Acid

**DOI:** 10.1021/acs.cgd.4c01715

**Published:** 2025-02-20

**Authors:** Stuart R. Kennedy, Toby J. Blundell, Elizabeth F. Henderson, Adeline P. Miquelot, Jonathan W. Steed

**Affiliations:** Department of Chemistry, Durham University, South Road, Durham DH1 3LE, U.K.

## Abstract

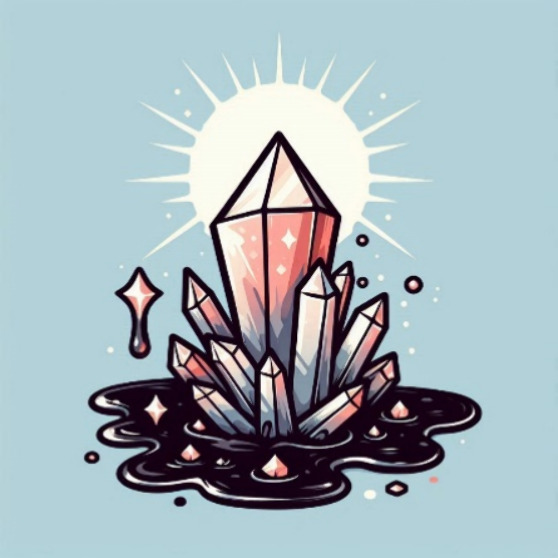

The salts of 5-ureidosalyclic acid (5-USA) form a range of pillared salt inclusion complexes from basic solvents, namely, triethylamine, piperidine, morpholine, and 4-ethylpyridine. The triethylamine derivative is unsolvated, while the other salts include 1–3 solvent molecules of formula XH^+^(5-USA-H)^−^·*n*X (X = morpholine, *n* = 1, 2, and 3 (**2a** – **2c**); X = 4-ethylpyridine, *n* = 1 (**3**) and X = piperidine, *n* = 2.5 (**4**)). The morpholine salt expands the layered structure to include 1, 2, or 3 guests. The 5-USA anion can also act as a bidentate ligand for manganese(III), again forming highly solvated inclusion complex anions [Mn(5-USA-H)_2_(Solv)_2_]^−^. When interacting strongly with the countercation, the 5-USA anions adopt an unusual *cis* carboxylate isomer form, whereas the morpholinium salt solvate (H-morpholine)[Mn(5-USA-H)_2_(morpholine)_2_]·2(morpholine) (**5c**) is *trans*. The soft, plastic crystals of this complex gradually desolvate, losing channel morpholine and ultimately spontaneously recrystallizing as the 5-USA salt dimorpholine inclusion complex **2b**. The complex hydrogen-bonded, pillared 5-USA salts illustrate a design concept for intercalated materials of forming charged, planar layers that leave the charge-balancing counterion exposed, such that interlayer guest species must be incorporated to effectively solvate the charged moieties.

## Abstract 2

Crystals of a urea−salicylic acid derivative form charged, planar layers that leave the counterion exposed causing incorporation of further interlayer guests. One such layered cocrystal crystallizes from desolvation and deliquescence of plastic crystals of a manganese complex of the urea−salicylic acid ligand.

## Introduction

A powerful concept in the design of inclusion compounds with controlled solid-state cavities is the formation of layered materials with well-defined spacers between the layers that are smaller than the layer width. This results in the creation of galleries for guest inclusion, with the gallery height being tunable by the spacer dimensions. This basic design is found in naturally occurring clay minerals and has been mimicked to excellent effect in the guanidinium monosulfonate (GMS) and guanidinium disulfonate (GDS) inclusion complexes.^1−5^ The occurrence of a reproducible layered motif with controllable linkages between the layers allows control over the interlayer spacing and gallery volume. As a result, the recurring layer motif can host varying amounts of guest for potential and actual applications in separations,^6^ selective crystallization of pheromones or luminophores,^7,8^ chiral separation,^9^ structure determination,^10^ nonlinear optical materials,^11^ or the study of guest dynamics and reactivity.^12^

Over 500 crystalline GMS and GDS porous crystals are known with lamellar, cylindrical and cubic architectures.^2^ While many early GMS structures are nonporous, the simple continuously layered inclusion compounds (s-CLIC) based on noncovalent layers of benzenesulfonates can include a range of guests.^13^ In the guanidinium sulfonates, the gallery height is controlled by the covalent substituent on the sulfonate group. An archetypical example of such a group is the *p*-phenylene spacer in guanidinium 1,4-benzenedisulfonate, which gives rise to a conventionally porous phase capable of absorbing a range of gases.^3^

In contrast to the covalent GDS compounds, for GMS layered solids there are fewer constraints on packing because of the absence of a covalent connection between the guanidinium sulfonate sheets. Such noncovalently connected materials can potentially swell to absorb more guest species and thus exhibit considerable versatility in guest sorption properties. Though the guanidinium sulfonates are perhaps the best explored examples, other layered materials, such as cation phosphonates, can also adopt porous pillared or zeotype structures with applications as heterogeneous catalysts in fine chemical synthesis.^14^

**Figure 1:**
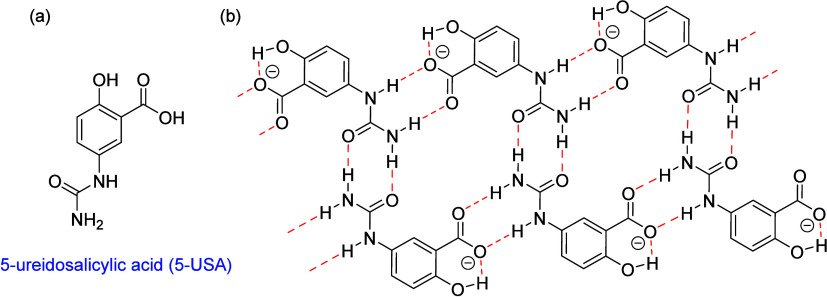
(a) Structure of 5-ureidosalicylic acid (5-USA). (b) Hydrogen-bonded ribbon formed by the deprotonated 5-USA anion.

In previous work we have reported the formation of planar, hydrogen bonded “cyclamer” type^15^ structures based on neutral 5-ureidosalicylic acid (5-USA) that are capable of including a range of guest species perching on the hydrogen bonded cyclic structure.^16^ We now report that, in the presence of bases, 5-USA is deprotonated to produce a range of pillared layer structures based on 1D ribbons of anionic hydrogen bonded rings (Figure 1). The interlayer separation of these structures is determined by the choice of countercation. The resulting salts exhibit versatile inclusion behavior, in one case expanding to form a series of salt cocrystals containing one, two or three guest molecules of the same type. We also report the extension of these systems to manganese(III) complexes of 5-USA including an unusual morpholine plastic crystal that decomposes to yield a 5-USA inclusion complex salt.

## Results and Discussion

### Ammonium Salt Layered Complexes

**Scheme 1:**
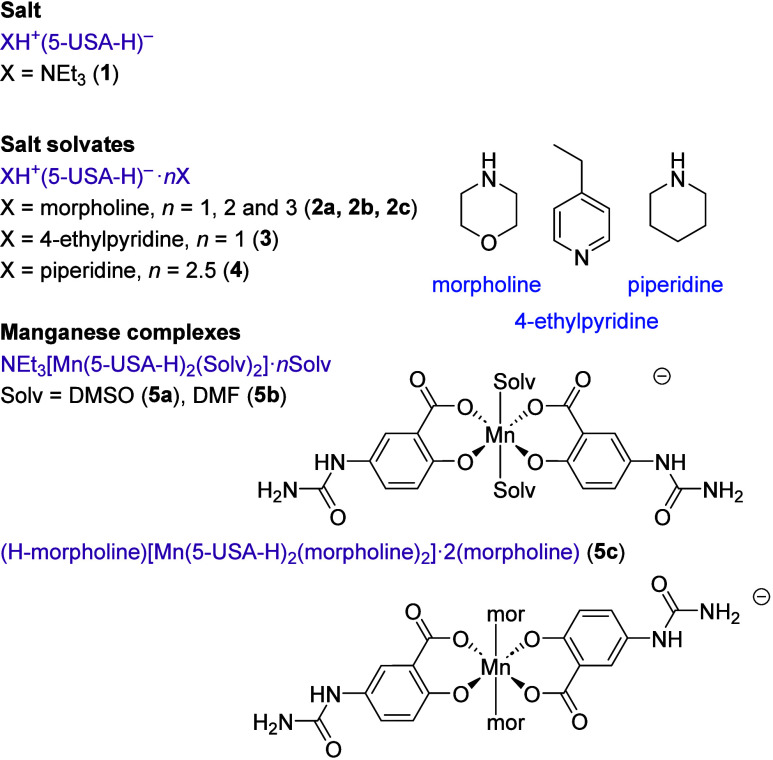
New Salts, Salt Solvates, and Manganese(III) Complexes Prepared in This Work

Crystallization of 5-USA from a range of basic solvents, namely triethylamine, piperidine, morpholine and 4-ethylpyridine, gives a range of salts and salt cocrystals of the 5-USA anion of type XH^+^(5-USA-H)^−^ for X = NEt_3_ (**1**), and XH^+^(5-USA-H)^−^·*n*X (X = morpholine, *n* = 1, 2, and 3 (**2a** – **2c**); X = 4-ethylpyridine, *n* = 1 (**3**) and X = piperidine, *n* = 2.5 (**4**)). The structures and formulas of all new materials are shown in Scheme 1. The dimorpholine salt cocrystal **2b** was discovered from an unusual decomposition of a manganese(III) complex, vide infra. The structures of salts **1**–**4** are all based on planar or distorted cyclic ribbon motifs of the type shown in Figure 1b. This structure is able to adapt to accommodate the organic cations and varying amounts of ordered neutral guest molecules. The morpholine complexes **2a**–**c** are remarkable in that the same morpholinium-5-USA salt motif is able to expand to cocrystallize with 1, 2, or 3 ordered molecules of neutral morpholine.

**Figure 2:**
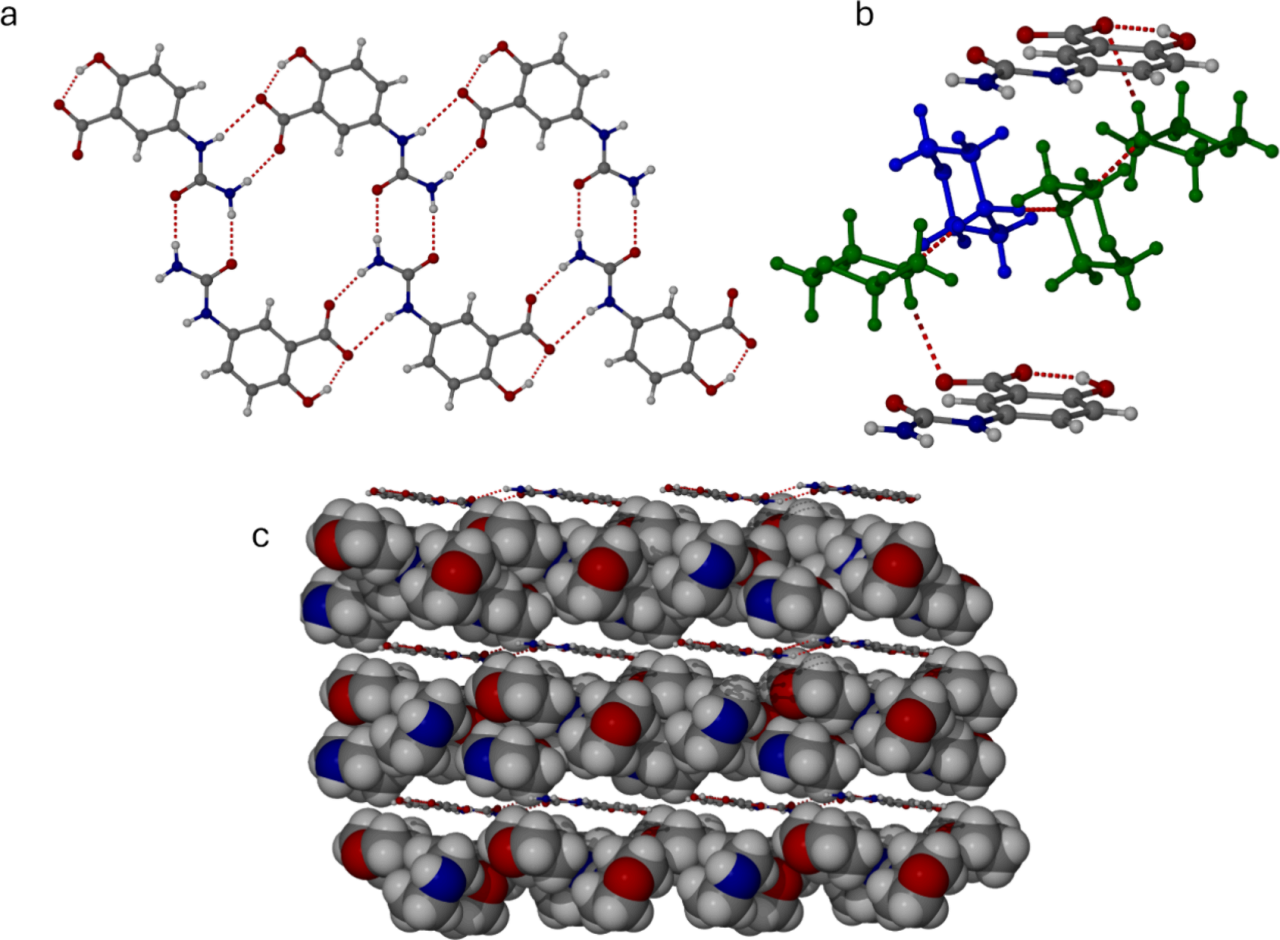
(a) 5-USA anionic layer in the morpholine trisolvate **2c**. (b) Detail of the protonated morpholinium cation (blue) and included morpholine guests (green) linked by NH···N hydrogen bonds and long NH···O interactions from the neutral morpholine guests to the anionic carboxylate oxygen atoms (N···O 3.07 and 3.16 Å). (c) Layered structure of **2c** (the 5-USA anion ribbons are shown in ball-and-stick representation, while the guest morpholine atoms and charge-balancing morpholinium cations are shown in space-filling mode).

The layered structure of the morpholine trisolvate **2c** is shown in Figure 2a. The anionic 5-USA ribbons are situated 9.21 Å apart with the layers stacked along the crystallographic *b* axis. The ribbons are situated edge-to-edge resulting in continuous, planar sheets of 5-USA anions sandwiching the morpholinium cations and morpholine solvent molecules. The morpholinium cation forms strong, charge-assisted hydrogen bonds to two independent morpholine amine lone pairs, N···N distances 2.69 and 2.83 Å. The third morpholine molecule receives a neutral hydrogen bond from one of the other morpholine molecules, N···N 2.98 Å. This longer hydrogen bond distance allows unambiguous identification of the protonated and neutral morpholine units. The result of this pattern of interactions is a discrete chain of morpholine moieties terminated at each end by secondary amine NH groups that form very long, out-of-plane NH···O interactions with the framework carboxylate groups, N···O 3.07 and 3.16 Å. The inclusion of solvent appears to arise from the strong driving force of providing hydrogen bond acceptors to the morpholinium cation NH groups, given that the carboxylate groups of the anionic framework are essentially unavailable for hydrogen bonding. Generally, hydrogen bonds involving charged groups are much stronger than those of neutral systems.^17^ Moreover, since only two morpholine molecules are required to act as hydrogen bond acceptors for the two NH_2_^+^ protons, the third morpholine molecule is adopting a space-filling role and is potentially redundant.

**Figure 3:**
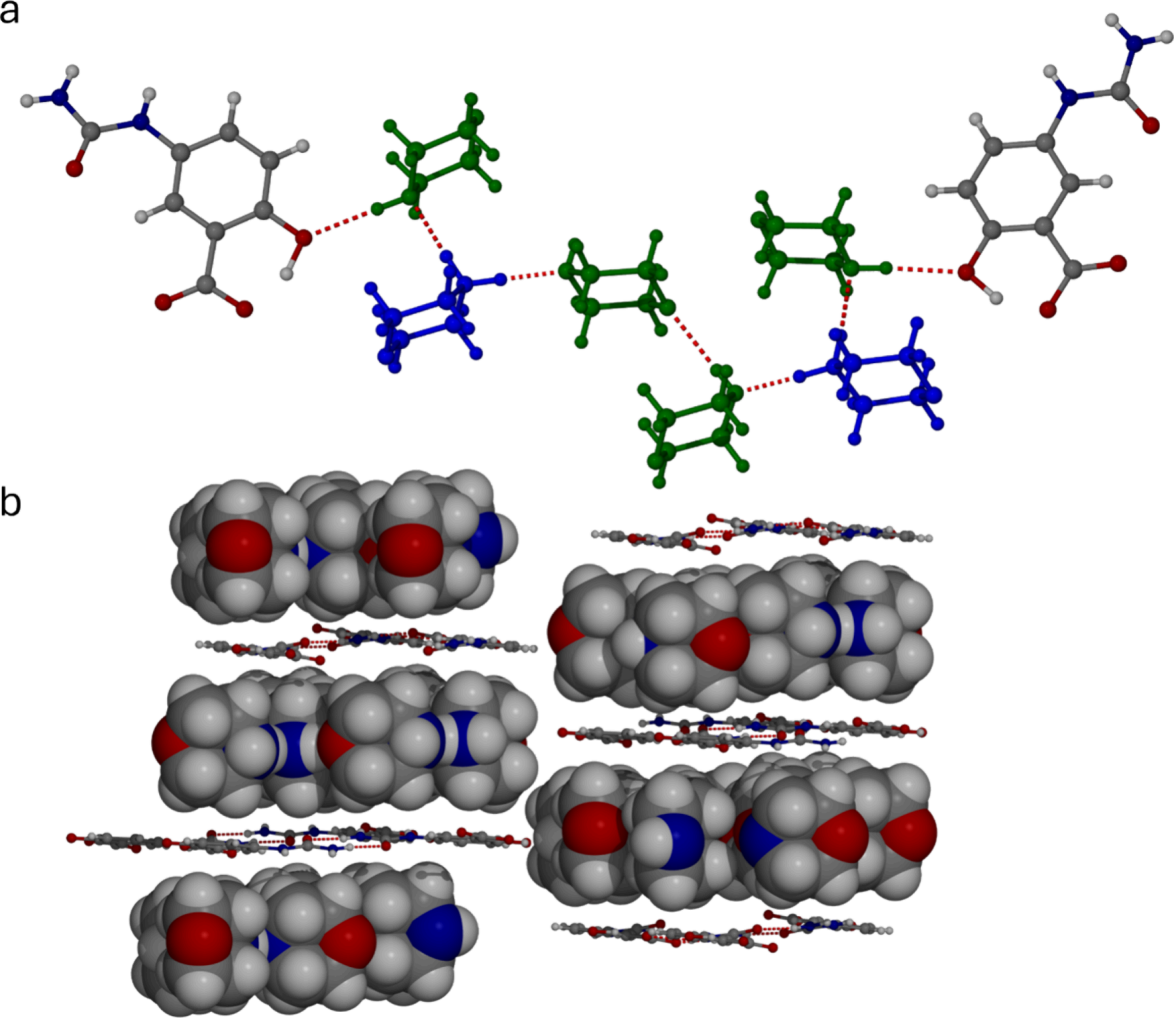
(a) Protonated morpholinium cation (blue) and included morpholine guests (green) in disolvate **2b** linked by NH···N hydrogen bonds (N···N 2.72 and 2.88 Å) and long NH···O interactions from the neutral morpholine guests to the anionic carboxylate oxygen atoms (N···O 2.98 and 3.06 Å) interactions. (b) Layered structure of **2b** (the 5-USA anion ribbons are shown in ball-and-stick representation, while the guest morpholine atoms and charge-balancing morpholinium cations are shown in space-filling mode).

Consistent with this hypothesis, the structure of the dimorpholine solvate **2b** retains the same pair of NH_2_^+^···N interactions between the morpholinium cation and two neutral morpholine acceptors (N···N 2.72 and 2.88 Å). This three-molecular unit is again terminated by neutral secondary amine NH groups, one of which forms a hydrogen bond to a morpholine oxygen atom and the other a long hydrogen bond to the edge of the anionic framework (N···O 2.98 and 3.06 Å, respectively). The layered packing arrangement is similar to **2c**, with an interlayer separation of 9.01 Å, but the layers are alternately offset and interwoven by half a layer spacing in order to accommodate the missing guest volume (Figure 3).

**Figure 4:**
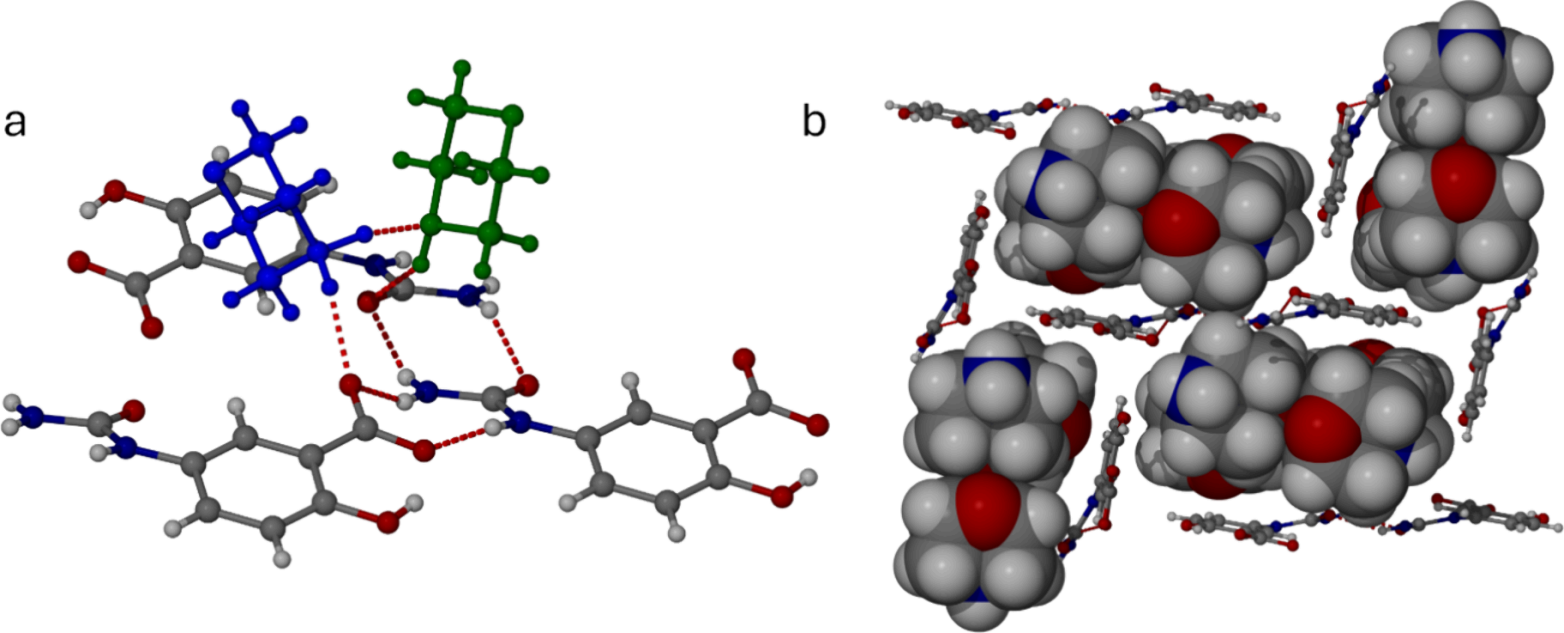
(a) Protonated morpholinium cation (blue) and included morpholine guest (green) in monosolvate **2a** linked by NH···N hydrogen bonds (N···N 2.76 Å) and NH···O interactions from the morpholinium cation to a 5-USA oxygen atom (N···O 2.76 Å) and from the neutral morpholine NH to a urea carbonyl group (3.11 Å). (b) 1D channels in **2a** arising from edge-to-face packing of the 5-USA anionic ribbons.

In the monosolvate **2a**, the morpholinium cation forms one NH···N interaction from the cationic NH_2_^+^ group to the neutral morpholine secondary amine as in **2b** and **2c** but it lacks a partner for the other of the cationic NH_2_^+^ hydrogen bond donors. As a result, the anionic framework distorts from planarity in order to allow one of the carboxylate oxygen atoms to act as a bifurcated acceptor, accepting a charged hydrogen bond from the cation (N···O 2.76 Å) while still taking part in the near-planar 5-USA ribbon structure. The neutral morpholine NH group forms a very long, perpendicular hydrogen bond to the urea carbonyl oxygen atom (N···O 3.11 Å). The further reduced guest volume is compensated by edge-to-face stacking of the pseudoplanar anionic ribbons to create 1D channels rather than 2D layers (Figure 4).

The isolation of the three morpholine solvates (**2**) allows the generalization that layered intercalate compounds such as **2c** may arise due to the tendency for a strongly hydrogen bonding cation to be surrounded by acceptors, in cases where the charge-balancing anionic framework is tied up in a layered structure and not freely available for hydrogen bonding. However, the system can adapt by increasing interweaving and distortion of the anionic layers if the cation is insufficiently surrounded by hydrogen bond acceptors.

**Figure 5:**
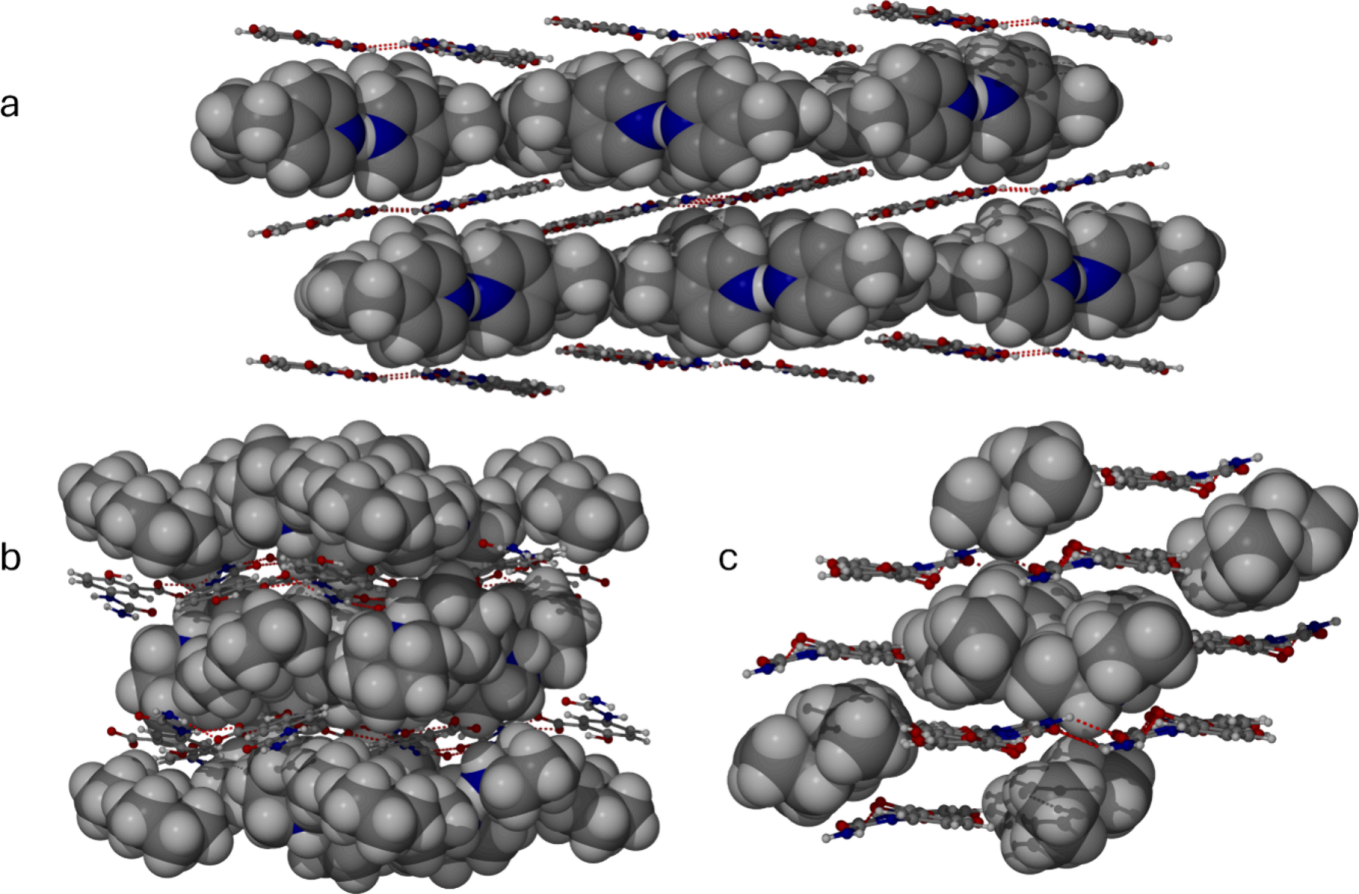
(a) Simple layered structure of the 4-ethylpyridine salt monosolvate **3**. (b) Complex layered structure of the piperidine salt hemipentasolvate **4**. Despite the low-symmetry distorted structure, it is closely related to **2c**. (c) Intercalated layer structure of the triethylammonium salt **1**.

Salt solvate complexes with other bases, namely the 4-ethylpyridine monosolvate (**3**) and piperidine hemipentasolvate (**4**), can be rationalized through the same lens. Solvate **3** contains one protonated solvent molecule, the hydrogen bonding requirements of which are satisfied by hydrogen bonding between the pyridinium NH^+^ group and a neutral 4-ethylpyridine acceptor. The solvated cationic unit intercalates between the same 5-USA anionic layers as the morpholine structures, and the irregular shape of the 4-ethylpyridine units causes the layers to slightly overlap and produce wedge-shaped interlayer vacancies (Figure 5a). Formation of a monosolvate is readily rationalized by the existence of a single pyridinium NH^+^ group.

In the piperidine complex **4,** the structure follows similar principles to **2c,** with continuous sheets of 5-USA anionic layers sandwiching piperidine-solvated piperidinium cations. However, the sheets adopt a wave-like distortion to accommodate the pendant neutral piperidine NH groups. There are two crystallographically independent 5-USA anions, one planar and one distorted, and both form the usual 5-USA ribbon structure. In addition, the planar anion interacts on one side with a hydrogen-bonded trimer of a piperidinium cation and two neutral piperidine molecules, which in turn form hydrogen bonds to the 5-USA framework. On the other side, the anion accepts a very long hydrogen bond to its OH group from a centrosymmetric H(piperidine)_2_^+^ cation. The distorted 5-USA anion interacts with a second, independent centrosymmetric H(piperidine)_2_^+^ cation, which hydrogen bonds at each end with the 5-USA carboxylate group.

The only nonsolvated 5-USA salt isolated in this study is the triethylammonium salt **1**. The structure is, however, entirely consistent with the architectural principles of the other members of the series. The layers distort, as in **2a**, to accommodate a direct NH^+^···O hydrogen bond from the cation to the 5-USA carboxylate group, while the large size of the cation gives rise to the kind of interwoven layers seen in the structure of **2b**.

### Manganese(III) Complexes

Given the ubiquity and robustness of the 5-USA anionic hydrogen bonded layer, we sought to disrupt its packing by metal complexation. In related cases, metal coordination of competing hydrogen bond acceptors can result in supramolecular metallogel formation.^18^ Metal complexes of salicylic acid derivatives are well-known, with the salicylate anion acting as a robust bidentate chelate ligand.^19−22^ However, no metal complexes of 5-USA have yet been reported. While a number of metal salts were investigated, only manganese(III) gave crystalline materials of interest in the context of this work.

**Figure 6:**
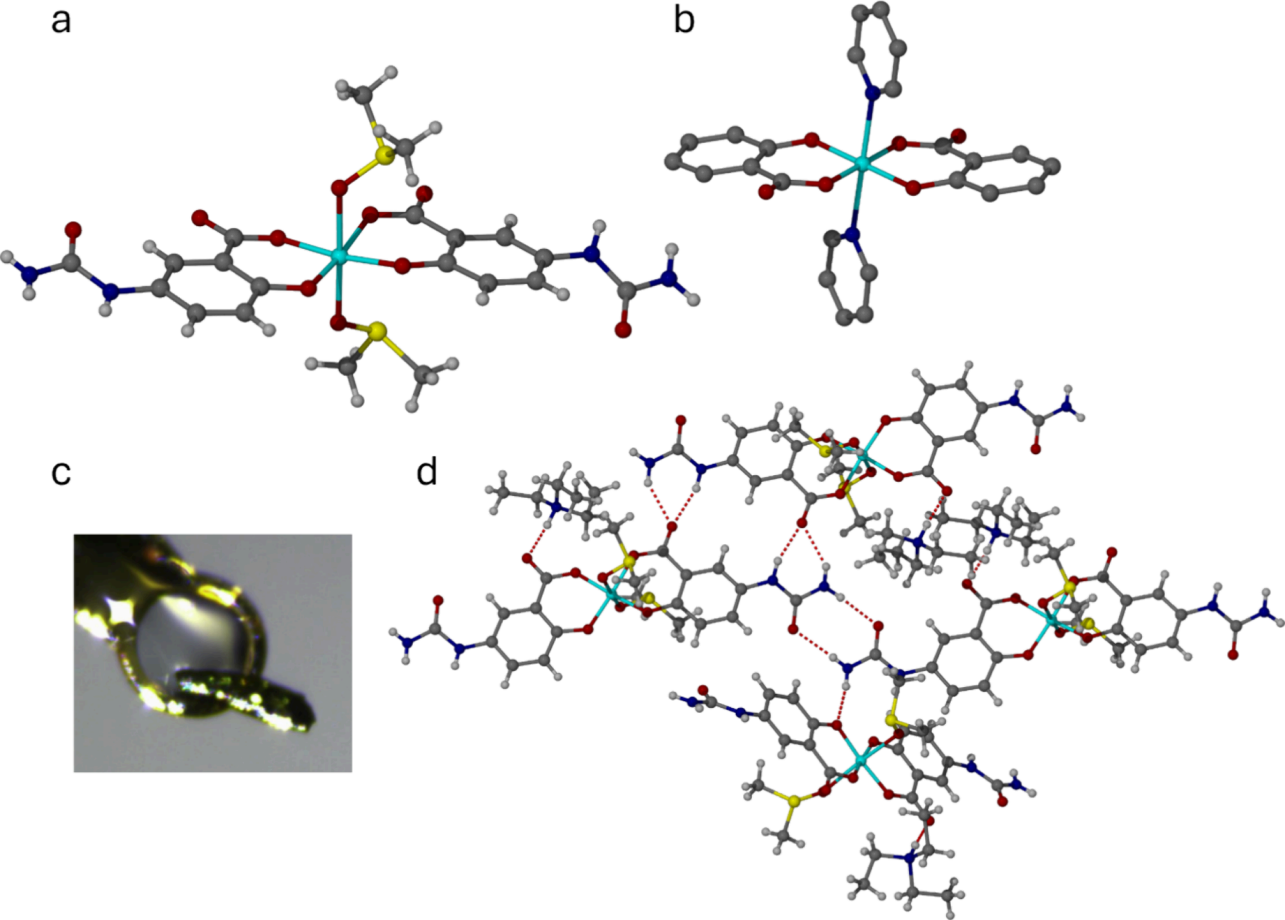
(a) Molecular structure of the *cis*-[Mn(5-USA-H)_2_(DMSO)_2_]^−^ anion in **5a** (the DMF complex **5b** is isomorphous). (b) Contrasting *trans* arrangement of the carboxylate groups in *trans*-[Mn(Salic)_2_(py)_2_]^−^ (NASWAF). (c) Mounted single crystal of **5a**. (d) Hydrogen-bonding pattern in **5a**.

When 5-USA was reacted with Mn(NO_3_)_2_·6H_2_O in air, alongside an excess of triethylamine to deprotonate the ligand, the metal underwent oxidation to produce dark black crystalline manganese(III) complexes of type NEt_3_[Mn(5-USA-H)_2_(Solv)_2_]·*n*Solv (where Solv = DMSO (**5a**) or DMF (**5b**)) (Figure 6a). The two complexes are isomorphous. The oxidation state of the manganese(III) centers, arising from aerobic oxidation of the manganese(II) starting material, is consistent with the observed dark color, the charge balance in the structure and the Jahn–Teller distorted nature of the high-spin, octahedral d^4^ metal centers^23^ observed in the X-ray structure determinations. In **5a**, for example, the equatorial Mn–O bonds range from 1.856(3) to 1.926(13) Å, while the axial bonds to the DMSO oxygen atom are 2.27 Å (average).

In both **5a** and **5b**, the carboxylate groups of the two 5-USA anions adopt a *cis* arrangement about the Mn(III) center. This feature is rarely observed in complexes where two untethered salicylic acid-derived ligands occupy the four equatorial or square planar sites of the metal center. The *trans* carboxylate arrangement, as in *trans*-[Mn(Salic)_2_(py)_2_]^−^ (CSD code NASWAF,^24^ Salic = salicylate, py = pyridine, Figure 6b), seems to be significantly more common, and would be expected on electronic grounds. The unusual structure of **5a** and **5b** appears to arise from electrostatic interactions of both carboxylate groups to the Et_3_NH^+^ cation.

Despite the perturbation introduced by the presence of the metal ion, the hydrogen bonding arrangement in **5a** and **5b** is somewhat related to the hydrogen bonded ribbon formed by the deprotonated 5-USA anion depicted in Figure 1b. In this case, however, the coordinated (anionic) carboxylate group accepts a hydrogen bond from the Et_3_NH^+^ cation and is unavailable to interact with the urea groups. The 8-membered hydrogen bonded ring motif formed by the urea self-association is retained, while one urea group forms an *R*_2_^1^(6) motif with one of the uncoordinated carboxylate oxygen atoms. The other urea forms a single hydrogen bond with a coordinated phenolate oxygen atom. The result is a distorted hydrogen bonded ribbon with coordinated solvent and pockets for uncoordinated solvent situated above and below the ribbon plane (Figure 6d).

When morpholine was used as the solvent and no triethylamine was added, a morpholinium salt (H-morpholine)[Mn(5-USA-H)_2_(morpholine)_2_]·2(morpholine) (**5c**) was isolated. Complex **5c** forms as large red-brown crystals over 24 h. The crystals deform readily and behave in a plastic fashion.^25,26^ Upon standing for 1 month, they undergo a remarkable decomposition to produce colorless crystals and a dark oil. The colorless crystals proved to be the disolvate **2b** discussed above. This unusual decomposition process is the only way in which **2b** could be prepared. Compound **5c** readily desolvates slightly above the 129 °C boiling point^27^ of morpholine. Warming the single crystals on the diffractometer gives rise to an initial small increase in unit cell volume followed by collapse of the structure.

**Figure 7:**
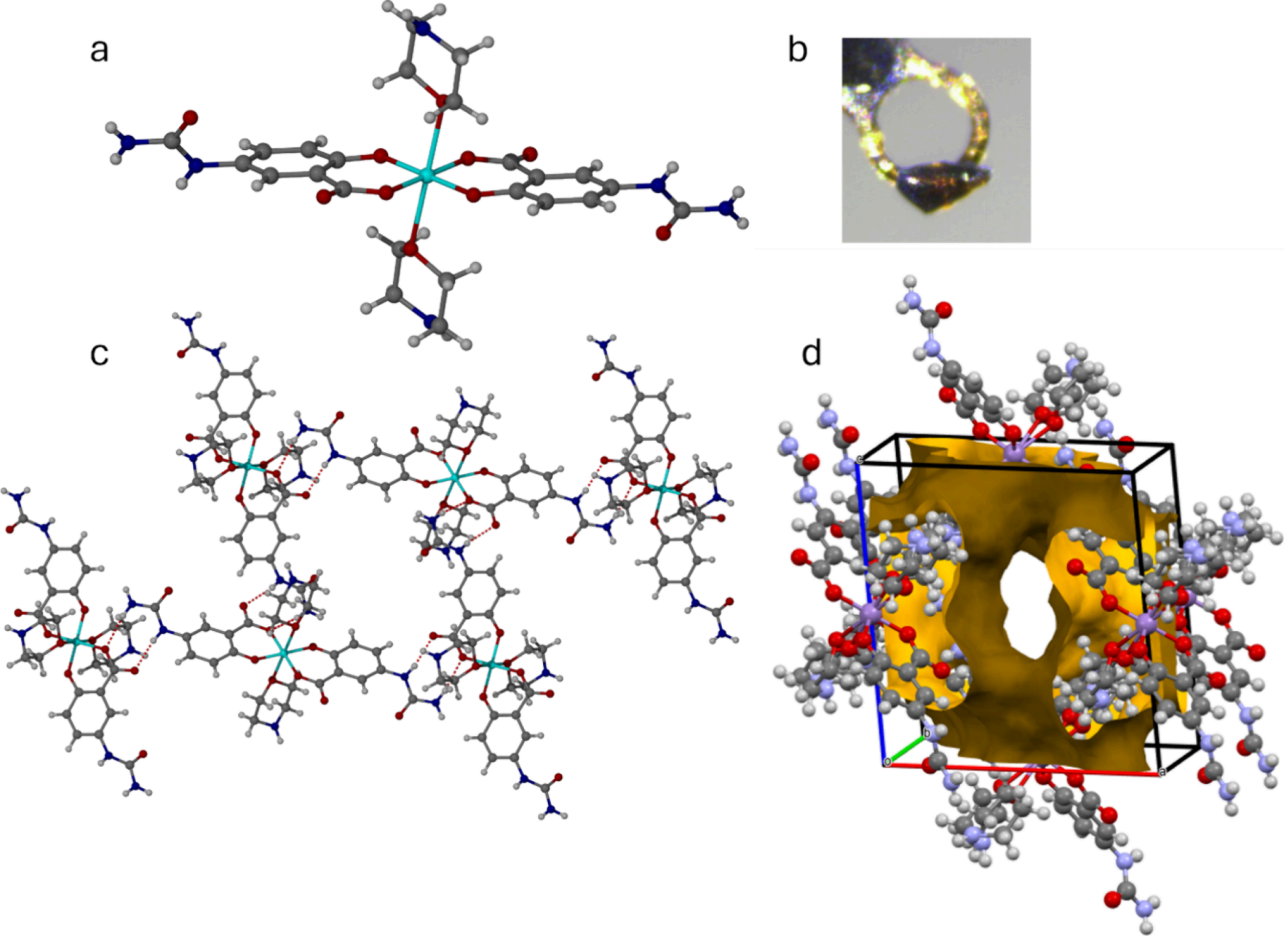
(a) Molecular structure of the *trans*-[Mn(5-USA-H)_2_(morpholine)_2_]^−^ anion in **5c**. (b) Mounted single crystal of **5c**. (c) Hydrogen-bonded macrocycle formation in **5c** based on *R*_2_^2^(8) urea-carboxylate interactions. (d) View of the 2D grid of morpholine-containing channels in **5c** that occupy 1083 Å^3^, or 46.9%, of the unit cell volume. Facile loss of morpholine from these channels results in plastic behavior and ultimately autodeliquescence and decomposition of the manganese complex to give a dark oil and crystals of **2b**.

Unlike **5a** and **5b**, the complex anion in **5c** is centrosymmetric and adopts a *trans* arrangement of the coordinated morpholine and carboxylate groups, comparable to the structure of NASWAF (Figure 7). This difference can be attributed to the presence of the morpholinium cation which, unlike the NEt_3_NH^+^ ion, does not form hydrogen bonds to the manganese complex. As in compounds **5a** and **5b,** the Mn(III) center is Jahn–Teller distorted with Mn–O distances of 1.865(3) (phenolate), 1.932(3) (carboxylate) and 2.380(10) Å (morpholine). The structure is based on hydrogen bonded macrocycles comprising four complexes, in which the urea and coordinated carboxylate groups are linked by *R*_2_^2^(8) ring motifs. These hydrogen bond arrangements are similar to the urea carboxylate interactions seen in Figure 2. The urea–urea 8-membered ring motif is absent, however, and the remaining urea NH protons interact with the nitrogen lone pair of the morpholine ligands.

It is remarkable that the extensive inclusion of morpholine by the 5-USA salt layers in **2a**–**c** described above also occurs in this 5-USA manganese complex. Due to the extensive disorder of all of the morpholine and morpholinium units, these molecules were treated using the PLATON SQUEEZE procedure.^28^ The lattice morpholine and morpholinium cation exist in a 2D grid of continuous channels along the crystallographic *a* and *b* directions, occupying 1083 Å^3^, or 46.9% of the unit cell volume (Figure 7d). The weakly bound, channel nature of this enclathrated morpholine solvent is likely to be the reason behind the deformable nature of the crystals and their facile loss of morpholine resulting, ultimately, in crystallization of the free ligand salt **2b**.

## Experimental Section

The 5-USA ligand was prepared according to the literature procedure.^15^

### Preparation of Salts

5-USA (5.0 mg, 0.026 mmol) was placed in a vial and 0.5 mL of basic solvent was added with a micropipette. The samples were then mixed, sonicated, warmed with a heat-gun and finally filtered before being allowed to stand at room temperature. Crystal formation occurred over several hours to a few days.

For the triethylammonium salt Et_3_NH(5-USA-H) (**1**), 5-USA (10.0 mg, 0.051 mmol) was dissolved in dimethylacetamide and sonicated to dissolved all of the solid material. Excess triethylamine (0.050 mL) was added and the mixture set aside for several weeks to give colorless crystals of **1**.

In the case of the morpholine salt solvate with *n* = 2 (**2b**), crystals formed from the dark black crystals of (C_4_H_10_NO^+^)[Mn(5-USA–H)_2_(C_4_H_9_NO)_2_]^−^·*n*(C_4_H_9_NO) (*n* ≈ 2) (**5c**), which deliquesce over a period of 1 month to give a dark liquid surrounding colorless crystals of (C_4_H_10_NO)^+^(5-USA-H)^−^·2(C_4_H_9_NO) (**2b**).

### Preparation of Manganese(III) Complexes

To prepare manganese complexes **5a** and **5b**, 5-USA (10.0 mg, 0.051 mmol) was dissolved in 0.5 mL of the relevant solvent (DMF, DMSO) and added to 0.5 mL of manganese(II) nitrate hexahydrate (9.4 mg, 0.033 mmol) in the same solvent. Excess triethylamine (0.050 mL) was added to give a transparent light brown solution, from which brown crystals deposited over periods of 1 day to 1 month. To prepare **5c**, 5-USA (5.0 mg, 0.026 mmol) and manganese(II) chloride (3.3 mg, 0.026 mmol) were added to 0.5 mL of morpholine and the mixture filtered to remove undissolved starting materials. Dark red-brown crystals of **5c** formed within a day.

## Conclusions

The anionic ribbon structure shown in Figure 1b is conserved in a remarkably consistent way across a wide variety of salts, leaving the polar, charge-balancing ammonium cations deficient in suitable hydrogen bond acceptors for their charged NH^+^ groups. This issue is mitigated by the inclusion of neutral hydrogen bond acceptor solvent and/or framework distortions while retaining the same fundamental layered structure. This interesting system illustrates a design principle for intercalated materials, whereby charged planar layers are formed to leave the charge-balancing counterion exposed and allow for the incorporation of further interlayer guest species.

The 5-USA anion also forms two interesting types of manganese(III) complex anions. In cases where the anion forms hydrogen bonds with the Et_3_NH^+^ cation, the carboxylate ligands adopt an unusual *cis* arrangement. In contrast, the highly solvated morpholine complex **5c** adopts the conventional *trans* geometry, leaving large morpholine-filled channels occupying almost half of the crystal volume. The facile loss of morpholine from these channels give the crystals a soft, plastic texture and, upon standing, results in decomposition and liquification of the manganese complex in what might be described as an autodeliquescence process. This solvent loss leads to recrystallization of the system as colorless crystals of the salt solvate (morpholinium)^+^(5-USA-H)^−^·2(morpholine), **2b**. The fact that the 5-USA anion delivers many solvates based on hydrogen bonded macrocycles, both as the free ligand and manganese(III) complexes, indicates a general propensity for the formation of these motifs.

## Supporting Information Available

X-ray crystallographic description and TGA data for **5b** (PDF)
