# Supplementary material for: Intercalation of Neutral Guests in Pillared Salt Cocrystals of 5-Ureidosalyclic Acid
Source: Cryst Growth Des. 2025 Feb 20;25(5):1614–21. doi: 10.1021/acs.cgd.4c01715 (PMC11887045; doi:10.1021/acs.cgd.4c01715)
Supplement: Supplementary file 1 — cg4c01715_si_001.pdf [file cg4c01715_si_001.pdf]

Intercalation of Neutral Guests in Pillared Salt Cocrystals of 5-Ureidosalyclic acid

Stuart R. Kennedy, Toby J. Blundell, Elizabeth F. Henderson, Adeline P. Miquelot and Jonathan W. Steed*

a) Department of Chemistry, Durham University, South Road, Durham, DH1 3LE, UK.

b)

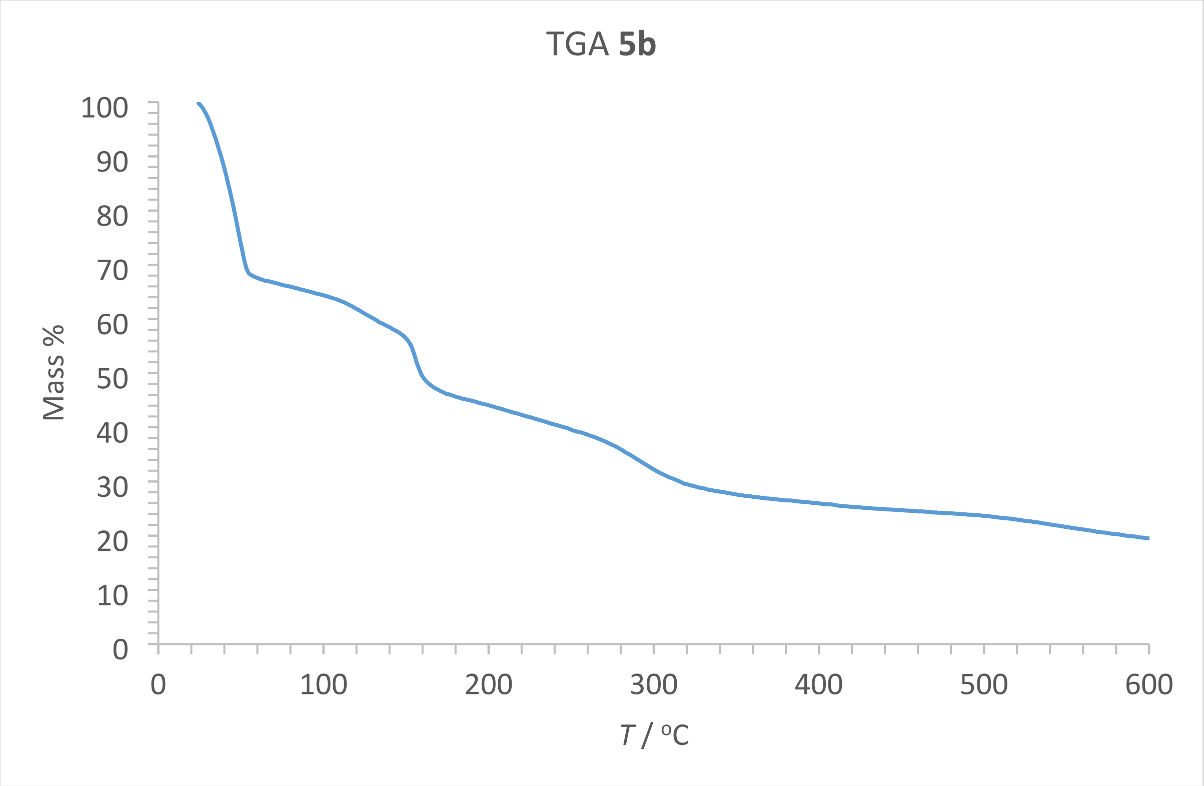

Fig. S1 TGA Thermogram for **5b**.

Single Crystal X-ray Crystallography

The X-ray single crystal data were collected using λMoKα radiation (λ =0.71073Å) on a Bruker D8Venture (Photon100 CMOS detector, IμS-microsource, focusing mirrors) 3-circle diffractometer equipped with a Cryostream (Oxford Cryosystems) open-flow nitrogen cryostat at the temperature 120.0(2)K. The structures were solved by direct method and refined by full-matrix least squares on F^2 for all data using Olex2 [1] and SHELXTL [2] software. All non-hydrogen atoms were refined in anisotropic approximation. The hydrogen atoms were placed in the calculated positions and refined in riding mode except where located by difference Fourier synthesis, in which case hydrogen atoms were refined whenever possible. Crystallographic data have been deposited with the Cambridge Crystallographic Data Centre as supplementary publications CCDC 2411206 – 2411213 **1**, **2a**, **2b**, **2c**, **3**, **5a**, **5b** and **5c**, respectively and 2421565 for **4**.

Table S1. Crystal data and structure refinement for **1**.

Identification code	nitmet2	
Empirical formula	C14 H23 N3 O4	
Formula weight	297.357	
Temperature	120.00 K	
Wavelength	0.71073 Å	
Crystal system	Triclinic	
Space group	P-1	
Unit cell dimensions	a = 8.2906(7) Å	α= 65.163(2)°.
	b = 13.6540(11) Å	β= 79.557(3)°.
	c = 14.6469(11) Å	γ = 83.136(3)°.
Volume	1478.0(2) Å^3	
Z	4	
Density (calculated)	1.336 Mg/m^3	
Absorption coefficient	0.099 mm^{-1}	
F(000)	640.455	
Crystal size	0.322 x 0.176 x 0.14 mm^3	
Theta range for data collection	2.50 to 26.50°.	
Index ranges	-11<=h<=11, -18<=k<=18, -19<=l<=19	
Reflections collected	21953	
Independent reflections	6118 [R(int) = 0.0650]	
Completeness to theta = 25.2417°	99.74 %	
Absorption correction	Semi-empirical from equivalents	
Max. and min. transmission	0.7457 and 0.6501	
Refinement method	Full-matrix least-squares on F^2	
Data / restraints / parameters	6118 / 0 / 421	
Goodness-of-fit on F^2	1.0541	
Final R indices [I>2sigma(I)]	R1 = 0.0701, wR2 = 0.1671	
R indices (all data)	R1 = 0.1260, wR2 = 0.2008	
Largest diff. peak and hole	0.9659 and -0.5642 e.Å^{-3}	

Table S2. Hydrogen bonds for **1** [Å and °].

D-H...A	d(D-H)	d(H...A)	d(D...A)	<(DHA)
N(203)-H(203)...O(201)	0.9300	1.964(3)	2.777(3)	145.00(9)
N(103)-H(103)...O(101)	0.97(3)	1.83(3)	2.734(3)	154(3)
O(203)-H(20c)...O(202)	0.90(4)	1.69(4)	2.552(3)	159(3)
N(201)-H(201)...O(102)	0.87(3)	2.11(3)	2.978(3)	178(3)
N(202)-H(20d)...O(101)	0.89(4)	1.90(4)	2.782(3)	171(3)
N(202)-H(20e)...O(204)#1	0.92(4)	2.05(4)	2.955(4)	168(3)
O(103)-H(10c)...O(102)	0.86(3)	1.75(3)	2.578(3)	158(3)
N(101)-H(101)...O(202)#2	0.87(3)	2.08(4)	2.948(3)	175(3)
N(102)-H(10d)...O(104)#3	0.91(4)	2.05(4)	2.948(3)	169(3)
N(102)-H(10e)...O(201)#2	0.90(3)	1.91(4)	2.803(3)	172(3)
C(107)-H(107)...O(104)	0.9500	2.181(3)	2.808(3)	122.53(9)

Symmetry transformations used to generate equivalent atoms:
#1 x+1,y+1,z+1 #2+0 #3 x,y,z+2

Table S3. Crystal data and structure refinement for **2a**.

Identification code	Mn2
Empirical formula	C16 H26 N4 O6
Formula weight	370.408
Temperature	120.00 K
Wavelength	0.71073 Å
Crystal system	Monoclinic
Space group	P 1 21/c 1

Unit cell dimensions	a = 8.5216(16) Å	α= 90°.
	b = 21.836(4) Å	β= 97.956(6)°.
	c = 9.5454(18) Å	γ = 90°.
Volume	1759.1(6) $Å^3$	
Z	4	
Density (calculated)	1.399 Mg/m^3	
Absorption coefficient	0.108 mm^{-1}	
F(000)	792.602	
Crystal size	0.164 x 0.118 x 0.093 mm^3	
Theta range for data collection	2.35 to 25.50°.	
Index ranges	-10<=h<=10, -26<=k<=26, -11<=l<=11	
Reflections collected	20366	
Independent reflections	3278 [R(int) = 0.0962]	
Completeness to theta = 25.2417°	99.94 %	
Absorption correction	Semi-empirical from equivalents	
Max. and min. transmission	0.7453 and 0.5998	
Refinement method	Full-matrix least-squares on F^2	
Data / restraints / parameters	3278 / 2 / 260	
Goodness-of-fit on F^2	1.0942	
Final R indices [I>2sigma(I)]	R1 = 0.0609, wR2 = 0.1527	
R indices (all data)	R1 = 0.1181, wR2 = 0.1924	
Largest diff. peak and hole	0.5888 and -0.5249 e.$Å^{-3}$	

Table S4. Hydrogen bonds for **2a** [Å and °].

D-H...A	d(D-H)	d(H...A)	d(D...A)	<(DHA)
O(3)-H(3)...O(2)	0.8400	1.795(3)	2.540(3)	146.9(3)
N(1)-H(1)...O(2)#1	0.97(4)	1.98(4)	2.949(4)	174(3)
N(2)-H(2a)...O(4)#2	0.90(3)	2.01(3)	2.909(4)	174(3)
N(2)-H(2b)...O(1)#1	0.88(3)	1.89(3)	2.766(4)	174(3)
N(11)-H(11a)...N(21)	1.06(4)	1.72(4)	2.766(4)	169(3)
N(11)-H(11b)...O(1)	0.91(3)	2.00(3)	2.768(3)	142(3)

Symmetry transformations used to generate equivalent atoms:
#1+0 #2 2

Table S5. Crystal data and structure refinement for **2b**.

Identification code	mo_A002A56	
Empirical formula	C20 H35 N5 O7	
Formula weight	457.530	
Temperature	120.00 K	
Wavelength	0.71073 Å	
Crystal system	Monoclinic	
Space group	P 1 21/c 1	
Unit cell dimensions	a = 16.748(3) Å	α= 90°.
	b = 8.3326(12) Å	β= 114.00(2)°.
	c = 17.905(3) Å	γ = 90°.
Volume	2282.6(8) $Å^3$	
Z	4	
Density (calculated)	1.331 Mg/m^3	
Absorption coefficient	0.101 mm^{-1}	
F(000)	984.729	
Crystal size	0.202 x 0.112 x 0.04 mm^3	
Theta range for data collection	2.30 to 25.00°.	
Index ranges	-19<=h<=19, -9<=k<=9, -21<=l<=21	
Reflections collected	25156	
Independent reflections	4004 [R(int) = 0.2544]	
Completeness to theta = 24.9974°	99.83 %	

Absorption correction	Sphere
Max. and min. transmission	0.93985 and 0.93985
Refinement method	Full-matrix least-squares on F^2
Data / restraints / parameters	4004 / 2 / 306
Goodness-of-fit on F^2	0.9962
Final R indices [I>2sigma(I)]	R1 = 0.0745, wR2 = 0.1525
R indices (all data)	R1 = 0.2227, wR2 = 0.2230
Largest diff. peak and hole	0.8385 and -0.8715 e.Å^{-3}

Table S6. Hydrogen bonds for **2b** [Å and °].

D-H...A	d(D-H)	d(H...A)	d(D...A)	<(DHA)
O(3)-H(3)...O(2)	0.8400	1.715(5)	2.472(5)	148.7(2)
N(2)-H(2a)...O(4)#1	0.8800	1.950(6)	2.827(6)	174.93(19)
N(2)-H(2b)...O(1)#2	0.8800	1.854(6)	2.728(6)	172.2(2)
C(7)-H(7)...O(4)	0.9500	2.159(7)	2.761(7)	120.14(18)
N(3)-H(3a)...N(21)	0.98(3)	1.75(3)	2.720(7)	169(5)
N(3)-H(3b)...N(11)	0.98(3)	1.88(4)	2.860(6)	180(5)
N(21)-H(21)...O(21)#3	0.90(5)	2.06(5)	2.949(6)	171(5)

Symmetry transformations used to generate equivalent atoms:
#1+4 #2 1 #3 x,y-1,z

Table S7. Crystal data and structure refinement for **2c**.

Identification code	a009mo1821	
Empirical formula	C24 H44 N6 O8	
Formula weight	544.65	
Temperature	120.00 K	
Wavelength	0.71073 Å	
Crystal system	Triclinic	
Space group	P-1	
Unit cell dimensions	a = 8.3605(5) Å	α= 85.979(2)°.
	b = 9.2147(6) Å	β= 80.208(2)°.
	c = 17.7313(11) Å	γ = 89.722(2)°.
Volume	1342.76(15) $Å^3$	
Z	2	
Density (calculated)	1.347 Mg/m^3	
Absorption coefficient	0.101 mm^{-1}	
F(000)	588	
Crystal size	0.482 x 0.373 x 0.2 mm^3	
Theta range for data collection	2.216 to 29.999°.	
Index ranges	-11<=h<=11, -12<=k<=12, -24<=l<=24	
Reflections collected	21931	
Independent reflections	7806 [R(int) = 0.0479]	
Completeness to theta = 25.242°	99.8 %	
Absorption correction	Semi-empirical from equivalents	
Max. and min. transmission	0.7462 and 0.6413	
Refinement method	Full-matrix least-squares on F^2	
Data / restraints / parameters	7806 / 1 / 379	
Goodness-of-fit on F^2	1.020	
Final R indices [I>2sigma(I)]	R1 = 0.0599, wR2 = 0.1309	
R indices (all data)	R1 = 0.1005, wR2 = 0.1480	
Extinction coefficient	n/a	
Largest diff. peak and hole	0.513 and -0.244 e.$Å^{-3}$	

Table S8. Hydrogen bonds for **2c** [Å and °].

D-H...A	d(D-H)	d(H...A)	d(D...A)	<(DHA)
O(3)-H(3)...O(2)	0.92(2)	1.69(2)	2.5543(17)	155(2)
N(2)-H(2A)...O(1)#1	0.84(2)	1.96(2)	2.7881(19)	167.0(18)
N(2)-H(2B)...O(4)#2	0.92(2)	2.06(2)	2.9691(19)	172.2(19)
C(7)-H(7)...O(4)	0.95	2.23	2.850(2)	121.9
N(21)-H(21A)...N(11)	0.946(16)	1.897(17)	2.8306(19)	168.7(17)
N(21)-H(21B)...N(31)	0.994(19)	1.705(19)	2.6965(18)	176(2)
N(31)-H(31)...N(41)	0.838(19)	2.15(2)	2.9802(19)	172.2(18)

Symmetry transformations used to generate equivalent atoms:
#1 x+1,y,z #2 -x+2,-y,-z+1

Table S9. Crystal data and structure refinement for **3**.

Identification code	A082A19	
Empirical formula	C22 H26 N4 O4	
Formula weight	410.476	
Temperature	120.00 K	
Wavelength	0.71073 Å	
Crystal system	Orthorhombic	
Space group	Pbca	
Unit cell dimensions	a = 15.9156(10) Å	α= 90°.
	b = 8.7057(6) Å	β= 90°.
	c = 29.9317(17) Å	γ = 90°.
Volume	4147.2(5) Å^3	
Z	8	
Density (calculated)	1.315 Mg/m^3	
Absorption coefficient	0.092 mm^{-1}	
F(000)	1745.185	
Crystal size	0.18 x 0.106 x 0.091 mm^3	
Theta range for data collection	2.56 to 25.10°.	
Index ranges	-18<=h<=16, -10<=k<=7, -35<=l<=35	
Reflections collected	22994	
Independent reflections	3688 [R(int) = 0.0569]	
Completeness to theta = 25.1013°	99.81 %	
Absorption correction	Semi-empirical from equivalents	
Max. and min. transmission	0.7452 and 0.6769	
Refinement method	Full-matrix least-squares on F^2	
Data / restraints / parameters	3688 / 201 / 278	
Goodness-of-fit on F^2	1.0776	
Final R indices [I>2sigma(I)]	R1 = 0.0459, wR2 = 0.0971	
R indices (all data)	R1 = 0.0763, wR2 = 0.1123	
Largest diff. peak and hole	0.3953 and -0.4149 e.Å^{-3}	

Table S10. Hydrogen bonds for A082A19 [Å and °].

D-H...A	d(D-H)	d(H...A)	d(D...A)	<(DHA)
O(3)-H(2)...O(2)	0.8400	1.8156(19)	2.5625(19)	147.22(12)
N(2)-H(1A)...O(4)#1	0.8800	2.037(2)	2.8990(19)	166.12(7)
N(2)-H(1B)...O(1)#2	0.8800	1.850(2)	2.713(2)	166.24(6)
N(21)-H(21)...N(11)	0.8800	1.786(2)	2.662(2)	173.16(8)
C(7)-H(7)...O(4)	0.9500	2.173(2)	2.809(2)	123.29(6)

Symmetry transformations used to generate equivalent atoms:
#1+4 #2 1

Table S11. Crystal data and structure refinement for **4**.

Identification code	15srv194	
Empirical formula	C41 H71 N9 O8	
Formula weight	818.07	
Temperature	100.0 K	
Wavelength	0.68890 Å	
Crystal system	Monoclinic	
Space group	P 1 21/c 1	
Unit cell dimensions	a = 18.0345(6) Å	α= 90°.
	b = 15.0281(6) Å	β= 103.577(3)°.
	c = 16.6275(7) Å	γ = 90°.
Volume	4380.5(3) $Å^3$	
Z	4	
Density (calculated)	1.240 Mg/m^3	
Absorption coefficient	0.082 mm^{-1}	
F(000)	1776	
Crystal size	0.08 x 0.07 x 0.001 mm^3	
Theta range for data collection	1.79 to 26.50°.	
Index ranges	-23<=h<=23, -19<=k<=19, -18<=l<=21	
Reflections collected	40575	
Independent reflections	9670 [R(int) = 0.0486]	
Completeness to theta = 26.50°	97.0 %	
Absorption correction	Semi-empirical from equivalents	
Max. and min. transmission	1.0000 and 0.8525	
Refinement method	Full-matrix least-squares on F^2	
Data / restraints / parameters	9670 / 0 / 576	
Goodness-of-fit on F^2	1.054	
Final R indices [I>2sigma(I)]	R1 = 0.0517, wR2 = 0.1395	
R indices (all data)	R1 = 0.0698, wR2 = 0.1492	
Largest diff. peak and hole	0.464 and -0.330 e.$Å^{-3}$	

Table S12. Hydrogen bonds for **4** [Å and °].

D-H...A	d(D-H)	d(H...A)	d(D...A)	<(DHA)
O(1)-H(1)...O(2)	0.86(3)	1.77(3)	2.5764(18)	156(2)
N(1)-H(1A)...O(2)#1	0.80(2)	2.20(2)	3.0057(19)	175.1(19)
N(2)-H(2A)...O(4A)#1	0.88(2)	2.05(2)	2.9312(18)	177(2)
N(2)-H(2B)...O(3)#1	0.86(2)	1.94(2)	2.805(2)	179(2)
O(1A)-H(1AA)...O(2A)	1.06(3)	1.50(3)	2.5129(18)	158(3)
N(1A)-H(1AB)...O(2A)#2	0.85(2)	2.17(2)	3.004(2)	168(2)
N(1A)-H(1AB)...O(3A)#2	0.85(2)	2.63(2)	3.2830(19)	135.0(19)
N(2A)-H(2AA)...O(4)#2	0.91(2)	2.08(2)	2.9854(18)	177(2)
N(2A)-H(2AB)...O(3A)#2	0.88(2)	1.94(2)	2.816(2)	170.2(18)
N(3D)-H(3DA)...O(3)	0.92	2.00	2.905(2)	168.1
N(3D)-H(3DB)...N(3D)#3	0.92	1.80	2.716(3)	177.5
N(3)-H(3B)...O(4A)	0.97(2)	2.04(2)	2.9638(19)	158.4(18)
N(3C)-H(3C)...O(3A)	0.97(3)	2.17(3)	2.973(2)	139(2)
N(3A)-H(3AA)...N(3C)	0.93(2)	1.95(2)	2.875(2)	170.5(19)
N(3A)-H(3AB)...N(3)	1.08(3)	1.66(3)	2.740(2)	173(2)
N(3B)-H(3BA)...O(1A)	0.97(3)	2.15(3)	3.111(2)	168(2)

Symmetry transformations used to generate equivalent atoms:
#1 -x,y+3/2,-z+3/2 #2 -x,y+3/2,-z+1/2 #3+3

Table S13. Crystal data and structure refinement for **5a**.

Identification code	Beth1	
Empirical formula	C27.5 H45.25 Mn1 N5 O10.75 S2.75	
Formula weight	761.063	
Temperature	120.00 K	
Wavelength	0.71073 Å	
Crystal system	Monoclinic	
Space group	C 1 2/c 1	
Unit cell dimensions	a = 37.060(2) Å	α= 90°.
	b = 8.3343(5) Å	β= 99.136(2)°.
	c = 23.7914(15) Å	γ = 90°.
Volume	7255.2(8) $Å^3$	
Z	8	
Density (calculated)	1.394 Mg/m^3	
Absorption coefficient	0.582 mm^{-1}	
F(000)	3209.622	
Crystal size	0.411 x 0.175 x 0.134 mm^3	
Theta range for data collection	2.20 to 25.83°.	
Index ranges	-45<=h<=45, -10<=k<=10, -29<=l<=29	
Reflections collected	43497	
Independent reflections	6994 [R(int) = 0.0929]	
Completeness to theta = 25.2417°	99.94 %	
Absorption correction	Semi-empirical from equivalents	
Max. and min. transmission	0.7453 and 0.6248	
Refinement method	Full-matrix least-squares on F^2	
Data / restraints / parameters	6994 / 2213 / 624	
Goodness-of-fit on F^2	1.0790	
Final R indices [I>2sigma(I)]	R1 = 0.0835, wR2 = 0.2306	
R indices (all data)	R1 = 0.1307, wR2 = 0.2659	
Largest diff. peak and hole	1.0246 and -1.1717 e.$Å^{-3}$	

Table S14. Hydrogen bonds for **5a** [Å and °].

D-H...A	d(D-H)	d(H...A)	d(D...A)	<(DHA)

N(1)-H(1bb)...O(12)	0.9300	1.829(6)	2.749(6)	169.88(19)
N(1)-H(1aa)...O(12)	0.9300	1.821(6)	2.749(6)	175.7(2)
N(21)-H(21)...O(22)#1	0.8800	2.05(2)	2.81(2)	144.8(8)
N(22)-H(22b)...O(22)#1	0.8800	2.13(4)	2.90(3)	145.7(8)
N(12)-H(12a)...O(24)#2	0.804(17)	2.16(3)	2.962(17)	172(7)
N(12)-H(12a)...O(24a)#2	0.804(17)	2.06(2)	2.864(11)	173(7)
N(12)-H(12b)...O(23)#3	0.807(16)	2.13(3)	2.863(12)	151(5)
N(12)-H(12b)...O(23a)#3	0.807(16)	2.22(3)	2.982(9)	157(5)
N(21a)-H(21a)...O(22a)#1	0.8800	2.027(15)	2.876(16)	161.5(7)
N(22a)-H(22d)...O(14)#4	0.8800	2.032(10)	2.904(10)	170.6(4)

Symmetry transformations used to generate equivalent atoms:
#1+3 #2 3 #3 -x+1,-y,-z+1 #4+2

Table S15. Crystal data and structure refinement for **5b**.

Identification code	A011Tb	
Empirical formula	C29.5 H45.5 Mn N7.5 O10.5	
Formula weight	728.172	
Temperature	120.00 K	
Wavelength	0.71073 Å	
Crystal system	Monoclinic	
Space group	C 1 2/c 1	
Unit cell dimensions	a = 36.7703(14) Å	α= 90°.
	b = 9.1765(4) Å	β= 101.646(1)°.
	c = 23.2069(9) Å	γ = 90°.
Volume	7669.3(5) Å^3	
Z	8	
Density (calculated)	1.261 Mg/m^3	
Absorption coefficient	0.404 mm^{-1}	
F(000)	3077.041	
Crystal size	0.689 x 0.53 x 0.186 mm^3	
Theta range for data collection	1.92 to 27.50°.	
Index ranges	-55<=h<=55, -13<=k<=13, -34<=l<=34	
Reflections collected	92674	
Independent reflections	8802 [R(int) = 0.0389]	
Completeness to theta = 25.2417°	99.94 %	
Absorption correction	Semi-empirical from equivalents	
Max. and min. transmission	0.7464 and 0.6732	
Refinement method	Full-matrix least-squares on F^2	
Data / restraints / parameters	8802 / 2469 / 628	
Goodness-of-fit on F^2	1.0641	
Final R indices [I>2sigma(I)]	R1 = 0.0745, wR2 = 0.2286	
R indices (all data)	R1 = 0.0831, wR2 = 0.2425	
Largest diff. peak and hole	1.6812 and -0.8805 e.Å^{-3}	

Table S16. Hydrogen bonds for **5b** [Å and °].

D-H...A	d(D-H)	d(H...A)	d(D...A)	<(DHA)
N(11)-H(11)...O(13)#1	0.75(4)	2.18(4)	2.922(3)	171(3)
N(12)-H(12a)...O(24)#2	0.8800	2.064(3)	2.922(3)	164.75(13)
N(21)-H(21)...O(22)#3	0.87(4)	2.02(4)	2.855(3)	160(4)
N(22)-H(22a)...O(22)#3	0.8800	2.213(4)	2.997(4)	148.19(9)
N(22)-H(22b)...O(14)#4	0.8800	2.027(3)	2.901(3)	172.30(11)
N(4)-H(4b)...O(12)	0.9300	1.905(8)	2.779(8)	155.9(3)
N(5)-H(5)...O(12)	0.9300	1.828(11)	2.749(11)	170.0(3)

Symmetry transformations used to generate equivalent atoms:
#1 -x,-y-1,-z   #2+2   #3+3   #4 3

Table S17. Crystal data and structure refinement for **5c**.

Identification code	A008Mo911	
Empirical formula	C32 H48 Mn1 N8 O12	
Formula weight	791.723	
Temperature	120.00 K	
Wavelength	0.71073 Å	
Crystal system	Monoclinic	
Space group	P 1 21/n 1	
Unit cell dimensions	a = 12.2661(8) Å	α= 90°.
	b = 14.1450(9) Å	β= 96.666(1)°.
	c = 13.4038(8) Å	γ = 90°.
Volume	2309.9(3) $Å^3$	
Z	2	
Density (calculated)	1.138 Mg/m^3	
Absorption coefficient	0.343 mm^{-1}	
F(000)	835.319	
Crystal size	0.268 x 0.223 x 0.146 mm^3	
Theta range for data collection	2.10 to 25.00°.	
Index ranges	-14<=h<=15, -17<=k<=17, -16<=l<=16	
Reflections collected	25396	
Independent reflections	4070 [R(int) = 0.0609]	
Completeness to theta = 24.9975°	99.85 %	
Absorption correction	Semi-empirical from equivalents	
Max. and min. transmission	0.7454 and 0.6985	
Refinement method	Full-matrix least-squares on F^2	
Data / restraints / parameters	4070 / 785 / 242	
Goodness-of-fit on F^2	1.0794	
Final R indices [I>2sigma(I)]	R1 = 0.0870, wR2 = 0.2604	
R indices (all data)	R1 = 0.1091, wR2 = 0.2874	
Largest diff. peak and hole	1.3432 and -0.8068 e.$Å^{-3}$	

Table S18. Hydrogen bonds for **5c** [Å and °].

D-H...A	d(D-H)	d(H...A)	d(D...A)	<(DHA)
N(2)-H(2)...N(12)#1	0.8800	2.132(15)	2.978(15)	161.1(4)
N(11)-H(11)...O(12)#2	0.8800	2.015(6)	2.814(5)	150.35(18)
N(12)-H(12a)...N(2)#1	0.8800	2.173(15)	2.978(15)	152.0(4)
C(23A)-H(23c)...O(14)#1	0.9900	2.183(11)	2.887(11)	126.8(4)

Symmetry transformations used to generate equivalent atoms:
#1+3 #2 x,y-1,z

References

1. O. V. Dolomanov, L. J. Bourhis, R. J. Gildea, J. A. K. Howard and H. Puschmann, J. *Appl. Cryst.* (2009), **42**, 339-341.

2. G.M. Sheldrick, *Acta Cryst.* (2008), **A64**, 112-122
